# Athletes’ perspectives on return to sport after anterior cruciate ligament reconstruction and their strategies to reduce reinjury risk: a qualitative interview study

**DOI:** 10.1186/s13102-024-00920-7

**Published:** 2024-06-14

**Authors:** Anne Fältström, Timmy Gustafsson, Nils Wärnsberg, Sofi Sonesson, Anna Hermansen

**Affiliations:** 1 Region Jönköping County, Rehabilitation Centre, Ryhov County Hospital, Jönköping, 551 85 Sweden; 2 Unit of Physiotherapy, Department of Health, Medicine and Caring Sciences, Linköping University, Linköping, Sweden; 3 Faculty of Medicine and Health Sciences, Linköping University, Linköping, Sweden; 4 Futurum - the Academy for Health and Care, Region Jönköping County, Ryhov County Hospital, Jönköping, 551 85 Sweden

**Keywords:** Content analysis, Reinjury, Second knee injury, Re-rupture, Contralateral rupture, Qualitative research, Interviews

## Abstract

**Background:**

Insights derived from athletes who have completed the final phase of rehabilitation and successfully returned to their respective sports after anterior cruciate ligament (ACL) reconstruction could potentially contribute to the enhancement of therapeutic strategies. Therefore, the aim of this study was to explore athletes’ experiences, thoughts, and behaviours of final phase rehabilitation and return to sport after ACL reconstruction and to describe their thoughts about the risk of reinjury.

**Methods:**

This qualitative interview study included individual semi-structured interviews with 15 athletes after ACL reconstruction. All athletes were aged between 15 and 35 years (median, 23 years), had returned to their preinjury contact sport at elite or recreational competitive level, rehabilitated with different physioterapists (working in hospital, primary care or sport clinics), and had undergone primary ACL reconstruction between 14 and 59 months (median, 23 months) before the interviews. Data were analysed using qualitative content analysis.

**Results:**

Analysis of the data resulted in the following 4 main categories related to athletes’ experiences of the return to sport process and their thoughts about the risk of reinjury: Athletes’ strategies for safe return to sport; Support during rehabilitation and return to sport; The rehabilitation journey was worthwhile to be able to play again; and Reinjury is beyond one’s control.

**Conclusions:**

Athletes described strategies for a safe return to sport after ACL reconstruction, emphasizing continuous increased load, not forcing return to sport, injury prevention exercises, and seeking support from professionals and coaches. Despite loving their sport, the athletes had mixed feelings about undergoing additional rehabilitation if reinjured. The athletes recognized the high reinjury risk, attributing it to fate. These findings enhance understanding of athletes’ return to sport experiences after ACL reconstruction, their strategies to minimize reinjury risk, which might help optimizing care for this patient group.

**Supplementary Information:**

The online version contains supplementary material available at 10.1186/s13102-024-00920-7.

## Background

Young patients who return to sport (RTS) after anterior cruciate ligament (ACL) reconstruction (ACLR), especially contact sports, have an increased risk of sustaining new knee injuries compared with those who do not RTS; 20–42% sustain a new (ACL) injury to their ipsilateral or contralateral knee [1, 2]. Subsequent ACL injuries could lead to adverse consequences such as decreased knee function, activity level and quality of life [3]. Yet, most patients aim to return to their previous sport after ACLR [4]. Comprehensive rehabilitation with a patient-centred approach is required to optimize function after ACLR [5]. However, inadequate rehabilitation facilities, limited knowledge and time constraints are potential barriers to optimal rehabilitation [6].

Patients’ experiences of the rehabilitation process must be considered for good quality care [6, 7]. Patients’ perspectives on rehabilitation and RTS might give new insights on how to improve treatment and support for patients to safely RTS, i.e. minimize the risk of reinjury. Previous studies on patients’ experiences of rehabilitation after ACLR have shown that patients were frustrated due to unfilled expectations of progress during the rehabilitation, loss of motivation, and they expressed fear of reinjury and a need for more support regarding personal goal setting [8, 9]. Qualitative studies have also described that the decision to RTS after ACLR was largely influenced by psychosocial factors (hesitancy, lack of confidence, loss of identity, a persistent sense of uncertainty regarding full recovery, and fear of reinjury) [10–16]. Athletes have expressed that graded exposure and progression to RTS after an ACL injury is important to manage fear of reinjury [15].

The final phase of rehabilitation is critical because the athlete often is discharged from healthcare and needs to take responsibility for the transition from supervised rehabilitation to full sport participation. This is the most vulnerable part of rehabilitation when the risk of a subsequent ACL injury is highest [17]. There is limited knowledge regarding the experiences of athletes who have undergone ACLR and the final phase of rehabilitation and RTS and their thoughts about the risk of reinjury. Increased understanding of the athlete’s perspective is important for healthcare providers, working at hospitals, primary care or at sport clinics, to understand, support and optimize the treatment for athletes in the RTS phase after ACLR. Therefore, the aim of this study was to explore athletes’ experiences, thoughts, and behaviours of the final phase of rehabilitation and RTS after ACLR and to describe their thoughts about the risk of reinjury.

## Methods

### Study design

A qualitative research design with individual semi-structured interviews was used for data collection and qualitative content analysis with an inductive approach was used for data analysis. Individual interviews were carried out to provide a rich understanding of the athletes’ experiences of the world described in their own words. A constructivist approach was applied to data analysis and interpretation considering the athletes’ unique experiences of the phenomenon [18]. The study was designed and reported in accordance with the Consolidated Criteria for Reporting Qualitative studies (COREQ) checklist (Supplementary material 1) [19].

### Participants and setting

Athletes active in knee strenuous contact sports at any competitive level before ACL injury, who had returned to their contact sports after ACLR were eligible for the study. A purposeful sampling strategy was used to achieve a varied sample of athletes based on geographical location, age, sex, type and level of sport, time since ACLR, various operating surgeons, and physiotherapists (working at hospital, primary care or sport clinics). Athletes aged between 15 and 35 years who had undergone a primary ACLR 1–5 years before inclusion were recruited. The athletes had to speak and understand Swedish, have participated in cutting and pivoting sports such as football, handball and floorball before the injury and returned to their preinjury sport at the time of the interview. Athletes with associated major knee injuries that had a profound effect on rehabilitation, such as posterior cruciate ligament injuries or severe injuries to the medial or lateral collateral ligaments of the knee treated surgically, were excluded. Different physiotherapists in the authors’ network in Sweden were contacted through verbal communication or e-mail and asked to participate in the recruitment of athletes (previous patients at their clinics). Seven physiotherapist were involved in the recruitment process, contacted eligible athletes by telephone and e-mail and provided with oral and written information about the study. After agreeing to participate, the athletes were contacted by the interviewers to answer background questions via a secure web-based survey system (esMaker, entergate) and to schedule the interview. Seventeen eligible athletes were contacted; 2 did not answer the web-based questionnaire and were therefore not contacted to schedule the interview. The athletes were given the option of face-to-face or virtual (facetime/telephone) interview. The interviews were conducted by one of 2 interviewers (TG, a physiotherapist with experience of ACLR rehabilitation and NW, a medical student with own experience of ACLR, both men) not involved in the athletes’ care. The athletes were informed about the profession of the interviewer. The athletes received both written and oral information about the study. Informed consent was obtained from all athletes to participate in the study, and parental or guardian consent was not required for individuals aged 15 and older, in accordance with Swedish law and regulations (2003:460) and the Swedish Ethical Review Authority. The study was approved by the Swedish Ethical Review Authority (Dnr 2021–04020).

### Data collection

Background information about the athletes and descriptive data included age, sex, occupation, time since ACLR, graft, sport level graded into elite (highest level in team sport) and recreational (lower competitive levels), and type of sport, were collected using a structured questionnaire distributed via esMaker. Also, the questionnaires International Knee Documentation Committee Subjective Knee Evaluation Form (IKDC-SKF) [20] and a Swedish version of the ACL-Return to Sport after Injury (ACL-RSI) scale [21, 22] were used to provide descriptive data of the athletes perceived knee function (IKDC-SKF) and athletes’ emotions, confidence in performance, and risk appraisal in relation to RTS (ACL-RSI). These data was not used in the content analysis. Further background information (sports history in general, how their injury occurred, rehabilitation and their current knee status) was collected using structured questions before the interview started.

An interview guide was used during the interviews (Supplementary material 2). The guide consisted of open-ended questions about athletes’ experiences, thoughts and behaviours of going through the final phase of rehabilitation, and their thoughts about the risk of reinjury and prevention of reinjury after RTS. The interview guide was constructed and discussed within the research group and tested in 2 pilot interviews. The pilot interviews were transcribed verbatim and discussed among the researchers. The preliminary interview guide included information on the risk of reinjury with a subsequent question where the athletes were asked to rank the most important prevention interventions. In the final version, this question was omitted because athletes had difficulties with ranking prevention interventions which limited their ability to speak freely. The pilot interviews were not included in the analysis.

Fifteen interviews were carried out after the pilot interviews, 3 face to face at the interviewer’s clinic, 7 over FaceTime, 5 by telephone and according to the athletes’ preferences and due to the distance to the clinic. All interviews were given a code, and all identifying data were removed from the presentation of the results to protect their identity. All interviews were audio recorded and no field notes were taken. The interviews were transcribed verbatim. The interviews lasted for a median of 35 min (range, 13–53 min). After including 15 athletes rich and comprehensive data had been collected through the interviews and no new information was forthcoming in the last few interviews. Therefore, we discontinued the recruitment. Data were collected from October to November 2021.

### Data analysis

Qualitative content analysis with an inductive approach was used according to Graneheim and Lundman [23]. This method was selected because we aimed to investigate the manifest content in line with the aim of describing the athletes’ perspectives in their own words.

The first author (AF, PhD, physiotherapist with extensive experience in ACLR rehabilitation and research), the senior author (AH, PhD, physiotherapist with experience in qualitative methodology), both women, and NW were mainly responsible for the analysis. First, the transcripts were read in their entirety to get a general sense of the content. Second, the transcripts were reviewed and meaning units were identified. Third, the meaning units were condensed. Fourth, the condensed meaning units were coded. These first steps were performed separately by AF and NW and discussed with AH for confirmation. Codes with similar context were sorted into subcategories and subcategories with a common core content were subsequently sorted into main categories. The categorization process was performed by AF, NW, and AH. The analysis was performed using an Excel sheet. Quotations capturing the essence of what had been said were selected to illustrate the different subcategories. The sorting process and the categories were then discussed between all authors (TG, NW, AF, AH, and SS; PhD, a physiotherapist with extensive experience in ACLR rehabilitation, research, and experience in qualitative methodology) until consensus was reached that the categories covered the data and reflected the athletes’ perspectives of final phase rehabilitation, RTS and the risk of reinjury. An example to illustrate the analysis process is presented in Table 1.

**Table 1:** Examples of the analysis process with meaning units, condensed meaning units, codes, sub-categories and main category

Meaning unit	Condensed meaning units	Code	Subcategory	Main category
You hear examples of horror. I think the last time it was the World Cup. You saw the injury list; they showed which players were missing, and then it’s like half of all those missing from the teams are because of ACL injuries, and then you wonder, how much they practice, how much injury prevention they do, but it still happens (I9)	Half of all those missing from the teams are because of ACL injuries despite much injury prevention it still happens	Many elite players are off due to an ACL injury despite a lot of prevention training	The risk of reinjury is high	Reinjury is beyond one’s control
It does not help to think too much about reinjury because it is a snap and once you sustain a reinjury, it will happen so fast and you will not realize what happened. That is my picture of it, so it will not help to think too much about sustaining a reinjury (I11)	Does not help to think too much about reinjury because it is a snap and once you sustain a reinjury	Does not help to think about reinjury because if it happens, it happens	If it happens, it happens	

Abbreviation; I, informant. 9 and 11 are code numbers of the interviews. Examples of meaning units, condensed meaning units, codes, subcategories and categories from content analysis of interviews with athletes with primary ACL reconstruction. ACL, anterior cruciate ligament

## Results

Fifteen athletes (9 women) aged 15–35 years participated in the study. Eleven competed at a recreational level and 4 were elite athletes. The median time since primary ACLR was 23 months (range, 14–59 months). A wide range in scores was reported for subjective knee function according to the IKDC-SKF and athletes’ emotions, confidence in performance, and risk appraisal in relation to RTS based on the ACL-RSI (Table 2).

**Table 2:** Background information about the participating athletes

Characteristic	Athletes (*n* = 15)
Age (years), median (range)	21 (15–35)
Sex, males/females, *n* (%)	6 (40)/9 (60)
Occupation	10 students, 5 workers
Time since ACLR (months), median (range)	23 (14 − 59)
Time from ACLR to return to full training with the team, median (range)	10 (7–18)
Graft, all autografts	10 hamstring tendon, 3 BPTB, 2 quadriceps tendon
Sport activity	10 football, 3 handball, 2 floorball
Sport level	4 elite, 11 recreational
ACL-RSI total score, median (range)	6.8 (1.0 − 9.8)
IKDC-SKF score, median (range)	86 (36 − 98)

ACLR, anterior cruciate ligament reconstruction; BPTB, bone-patellar tendon-bone; ACL-RSI, ACL-Return to Sport after Injury scale, each question score range from 1 (worst) to 10 (best) in the Swedish version and the total score is the sum of all 12 questions divided by 12 (range 1–10) [21]; IKDC-SKF, International Knee Documentation Committee Subjective Knee Evaluation Form, score range from 0 (worst) to 100 (best)

The analysis resulted in 4 main categories: Athletes’ strategies for safe return to sport; Support during rehabilitation and return to sport; The rehabilitation journey was worthwhile to be able to play again; and Reinjury is beyond one’s control. The main categories were supported by 2–5 subcategories (Table 3).

**Table 3:** Overview of the main categories and subcategories

Main category	Subcategory
Athletes’ strategies for safe return to sport	Continuous increase in activity
	No stress to return
	My knee, my responsibility
	Importance of continuing with preventive training
	Avoiding situations because of fear of injury
Support during rehabilitation and return to sport	Prerequisites for efficient final phase rehabilitation
	Healthcare professionals provide guidance and calm
	Insufficient support from physiotherapists and coaches
The rehabilitation journey was worthwhile to be able to play again	Love of the sport is an incentive to return to sport
	If it happened again, I know what I must go through
Reinjury is beyond one’s control	The risk of reinjury is high
	If it happens, it happens

### Athletes’ strategies for safe return to sport

The athletes described a number of strategies to safely return to sport and to prevent further injuries. The strategies were that they emphasized continuous progress in returning to sport without stress to RTS and the fact that they were most responsible for their return process. They were aware of that they should continue preventive training after RTS, but it was difficult to maintain after they RTS. They also avoided certain situations to possibly alter the risk of reinjury.

#### Continuous increase in activity

All of the athletes reported a stepwise progression back into their sport following surgery. In practice, they had started with alternative training, then running, then running with the ball and so on, and during games they had initially played for a restricted time. Athletes described wanting more “match play in training” before playing a real match, because of the big difference between training and match play. They also stated that it is a big step either physically or mentally to go from rehabilitation to RTS.

‘*In August, I started attending the team sessions and warmed up and stuff. Then I was present until February when I played my first real game. So, I kind of increased my involvement step by step each week*’. *(I14)*

#### No stress to return

The athletes described that it was important to avoid stress during rehabilitation, and really take time to be able to RTS and be on the safe side i.e. not returning too early. They believed that time after reconstruction and returning to sport too early were important for the risk of reinjury and that patience with rehabilitation was required.

‘*I know many female football players who have ruptured their ACL two, sometimes three times, and what has been recurring in all those cases is that they returned too early*’. *(I2)*

#### My knee, my responsibility

The athletes reported that they were ultimately responsible for their own knee health and that the RTS process is a phase in rehabilitation for which they need to take responsibility. Some athletes expressed that their behaviour was the most important factor in preventing reinjury.

‘*I bear the main responsibility since I’m the one, or the athlete’s the one supposed to exercise and do the rehab and have the patience and the discipline to do so. So mostly, it’s up to me because if I don’t want to or if I’m not motivated or don’t want to become a 100% good again (restored knee function)*’. *(I4)*

#### Importance of continuing with preventive training

Athletes continued with preventive training to different extents. Some described the importance of continuing strength and rehabilitation exercises to avoid putting too much strain on the reconstructed knee or the healthy knee. Athletes also described that they neglected the injury prevention exercises they did during rehabilitation due to lack of motivation, lack of need or lack of time. They expressed that this noncompliant behaviour is detrimental.

‘*Now, after my injury, I’ve heard that strength training, extra strength training, is important in order to stay strong because I’ve heard that a weak knee also increases the risk (of reinjury). The more I, well I shouldn’t overdo it (the rehabilitation), but I must, or I try to exercise once a week focusing on my legs in order to prevent (a reinjury)*’. *(I14)*

#### Avoiding situations because of fear of injury

The athletes sometimes avoided situations at practice or during a game, and adapted behaviours in their daily life and became more cautious because of the fear of reinjury. They described that they dropped out in the middle of a training session or a game, reluctant to involve themselves in duels or sliding tackles; they stayed back, and instead of running at full speed and changing direction rapidly, they described they took some extra steps. However, being too cautious was thought by some athletes to be a risk for injury. They also described that as time went by, the fear disappeared little by little.

‘*I didn’t think I was afraid, but you noticed rather quickly that you were probably more afraid than you thought. I was constantly thinking about how I should put my knee down, so that it wouldn’t hurt and you ran around and felt, does it hurt now? … now I felt a little in my knee … what was happening … so even if I trusted my knee, you were probably still a little on your guard*’. *(I8)*

### Support during rehabilitation and return to sport

The athletes reported different ways in which they did and did not receive the support needed to RTS and minimize the risk of reinjury. They experienced that the physiotherapists offered guidance in rehabilitation, but there was a lack of support from coaches on how and when to return to high-intensity practice and games.

#### Prerequisites for efficient final phase rehabilitation

Athletes expressed that they needed someone with knowledge about ACL injuries to talk to because mentally, RTS was a big step and many lacked understanding of what rehabilitation after an ACLR implied. The athletes expressed that there was a lack of facilities for alternative training (gym, cycling) alongside the pitch to increase their sense of team affiliation when they could not participate in all stages of the usual training.

‘*So really, you would have liked to have someone there all the time who could guide you a little, because it was a bit difficult to judge by yourself all the time when it was too much or can I do this*’ *… (I8)*.

#### Healthcare professionals provide guidance and calm

The athletes expressed that the physiotherapists played a major role in guiding them during the rehabilitation process, and they felt that they could rely on them. They appreciated the sensible advice from the physiotherapists and their encouragement to continue with rehabilitation to become strong, continue being strong and prevent future injuries. The athletes stressed the importance of a follow-up consultation with the physician to confirm that everything was in order. This calmed athletes who thought that maybe something new and undesirable had occurred.

‘*There will be setbacks that you can’t predict. Like sometimes it (the knee) gets swollen and sometimes it hurts and sometimes it feels like you’ll never be able to return to your sport, and then that’s where the physiotherapist can help you and tell you it is normal and that it is not at all strange. Because this type of injury is also very psychological*’. *(I11)*

#### Insufficient support from physiotherapists and coaches

The athletes wished for more support from physiotherapists and coaches at the time of RTS. The athletes sought help from teammates, films on YouTube or parents to facilitate RTS when they did not receive enough information from the physiotherapists or coaches. From the athletes’ point of view, they expressed that they did not receive knowledge-based guidance and understanding from the coaches. They expressed that more knowledge and education about ACL injuries and rehabilitation after ACLR (understanding of the seriousness of the injury, time to RTS, to not stress the player) among coaches should facilitate the RTS process. The athletes also expressed that communication between the player, physiotherapist and coach is important to facilitate athletes’ experiences of a smooth RTS. Some athletes expressed that their coaches tended to push them harder than they felt comfortable with, whereas the physiotherapist wanted to wait/hold back the player. They believed that coaches need to respect the player’s (with ACLR) decision if he/she was ready to play or not. The athletes were not comfortable risking their knee health.

‘*The hardest part is how to relate to your coach. He is not educated (in sports medicine) so he does not know anything about rehabilitation, so how much should I listen to him in this situation?*’ *(I9)*.

### The rehabilitation journey was worthwhile to be able to play again

This category includes the athletes’ expression of their love for their sport and the goal to RTS served as motivation for completing the rehabilitation process. However, the athletes had mixed feelings about going through a further rehabilitation process if they sustained a reinjury. The thought of having to go through the process another time could be either discouraging, or bearable since it was already mastered once.

#### Love of the sport is an incentive to return

The athletes expressed a great love for their sport, which made the struggle to get back bearable. They were grateful for the opportunity to play again, as they have come to realize that playing is not something that can be taken for granted. If they had not RTS, they would have regretted it, as it is something they can do now and may not be able to do later on

‘It has been such a joy to be able to play football again and you appreciate football much more now after the injury than before because it is not something you can take for granted’. (I3)

#### If it happened again, I know what I must go through

The idea of what the rehabilitation process entails was both positive and negative for the athletes. Some felt that it was discouraging to think of the process they would have to go through if it happened again. Others felt that the experience from the first rehabilitation process was empowering. The fact that they had gone through it once made the prospect of going through a second rehabilitation more feasible because they now knew what lay ahead.

‘*I’m not running around worrying about reinjuring my ACL, but if it happened it would of course be really difficult because I know what I have to go through in order to get back*’. *(I1)*

### Reinjury is beyond one’s control

This category includes awareness among the athletes of the high risk of reinjury after ACLR and the experience that reinjury is due to chance and cannot be fully prevented.

#### The risk of reinjury is high

Athletes were aware of the high risk of reinjury to either knee. Awareness was based on people in their vicinity who had sustained a second injury or through information from medical staff.

‘*Was I really ready (to RTS)? It was a little scary and I knew that the percentage risk of injuring the same or the other knee again is way higher (compared with the likelihood of injuring your knee the first time)*’. *(I4)*

#### If it happens, it happens

Some athletes expressed that there was no point in worrying about reinjury following RTS because worring would not prevent reinjury and injuries continue to occur despite the athlete’s best efforts to prevent them. The athletes expressed that they tried to not think about reinjuries. They believed that reinjuries could occur through bad luck or because of fate and it was difficult to know what to do to prevent a reinjury.

‘*I’m more afraid that I’ll get unlucky and sustain an injury or it’s clear that there’s a risk that I could sustain an injury because the knee is weak, but I’m more afraid that just an accident will occur*’. *(I15)*

## Discussion

This study highlights the athletes’ experiences during final phase rehabilitation and RTS and their thoughts about risk of re-injury. The study contributes to increased understanding of the athletes’ perspectives of the process of returning to cutting and pivoting sports. The athletes’ experiences were summarized into 4 categories: Athletes’ strategies for safe return to sport; Support during rehabilitation and return to sport; The rehabilitation journey was worthwhile to be able to play again; and Reinjury is beyond one’s control.

Athletes expressed several strategies to safely RTS after the ACLR and to continue playing without sustaining new injuries, such as a continuous increase in activities, being patient, not stressing about RTS and continuing preventive training. Not stressing about RTS after ACLR [17, 24, 25] and continuing preventive training [25] have previously been reported as important factors in minimizing the risk of reinjury. Some athletes in the present study had stopped doing the preventive exercises due to a lack of motivation even though they thought it was valuable to reduce the risk of reinjury. It might be challenging to keep up injury prevention training after full RTS. Being confident and believing that the RTS will be successful has been stated previously as important to maintain a high level of motivation to continue with prevention exercises [8].

The athletes described that they aimed for a continuous increase in activities, which is in line with current rehabilitation guidelines [25, 26]. In addition to gradually building physical strength, results from a previous interview study showed that a progressive process from rehabilitation exercises to sports is important to rebuild confidence and manage fear of reinjury [15]. Athletes in the present study revealed that they sometimes avoided situations during games or game-like drills because of fear of reinjury. A high level of fear of reinjury is associated with increased reinjury risk [27]. Avoidance is a common coping strategy for fear of injury in athletes with ACLR, although that often leads to reduced quality of life [11, 28].

The athletes appreciated the guidance they received from their physiotherapist, although they expressed that they wanted more support from both physiotherapists and coaches at the time of RTS. The athletes wanted to have more sport specific exercises to do on the pitch, someone to discuss what exactly you can do on the pitch and what to expect in the progress, and someone who look after you to continue with rehabilitation exercises. The need for support and guidance has been reported previously in qualitative studies about the RTS process after ACLR [8, 9, 12, 14]. A previous study showed that athletes appreciate physiotherapists’ expertise, considering them important for providing knowledge, developing confidence, and giving reassurance [15].

The athletes did not have trust in the coaches’ knowledge about ACL injuries and rehabilitation after ACLR. This result indicates that coaches might need basic education about ACL injuries and athletes might need more support from medical personnel during the final phase of rehabilitation and RTS. This is in agreement with previous research showing that healthcare professionals, coaches, teammates, and family are important in giving support in the rehabilitation process after ACLR [11, 14]. The athletes emphasized the need for good communication between the athlete, physiotherapist, and coach. Good communication entailed consensus between the athletes, physiotherapist, and coaches when to be able to perform different tasks in the sport. Female elite football players have expressed that poor communication between players, healthcare professionals, and coaches was considered to be a barrier to successful RTS [29]. Furthermore, to enhance rehabilitation outcomes, healthcare professionals must cultivate a robust foundation of knowledge, proactively participate in the rehabilitation journey, and foster constructive communication with athletes. Essential to this is the ability to be accessible, engage in open dialogue, and inquire with thoughtful follow-up questions to gain a deeper comprehension of the athletes’ needs [14]. The importance of a trusting relationship with the coach during the rehabilitation process has previously been reported by high school level players [12].

In the present study, athletes wanted to be able to take part in alternative training close by the team to increase their sense of team affiliation, a result in line with a previously reported finding of feeling isolated when not participating in training with the team [9]. Social connections and support might serve as a reminder that the athlete is still a valuable team member [15] and a competitive rehabilitation environment may increase motivation to RTS [11]. In contrast to this result, female patients who have rerupture their ACL graft experienced that it was unbearable to stay close to their team and not being able to participate [30].

The athletes pointed out that the rehabilitation journey was worthwhile to be able to play again due to the love of their sport. Athletes with ACLR have previously expressed that a sense of athletic identity motivated them through the rehabilitation process [11, 15]. Being injured could have a great impact on young (< 22 years) athletes’ identity and could increase the risk of depression [31]. Some of the athletes in the present study felt prepared to undergo rehabilitation again if they sustained a reinjury. Mental toughness and commitment to oneself as well as one’s team and sport have been described as drivers for RTS in athletes after ACLR [15].

Reinjuries were considered common and thought to often occur due to fate. Previously, athletes who had sustained an ACL rupture considered their injury was due to an accident, rather than based on intrinsic risk factors [15]. The athletes in the present study thought that there was no use worrying about reinjury and argued that “if it happens, it happens”. These thoughts are somewhat in contrast with the strategies described for a safe RTS and to reduce the risk of reinjury. These contrasting findings might indicate that the athletes knew that rehabilitation was their only way to RTS even though they could not fully control not being injured again.

Understanding the athletes’ experiences and thoughts in the final phase of rehabilitation may help healthcare professionals identify factors that could contribute to optimizing the care in this patient group. The clinical implications of our study are that the physiotherapists should carefully structure the rehabilitation with continuous increased loads, schedule follow-up consultations focusing on both physical and mental needs, as well as support the athlete to not stress about RTS and continue with long-term use of preventive training [14, 17, 25, 26]. A sport psychologist may be contacted in same cases to address mental and behavioral needs [32]. The healthcare professionals (surgeons and physiotherapists) should strive for good communication with the athlete and the coach aiming for an optimal individual workload for the athlete [14, 29]. Information and support for coaches should be provided to increase the ability of the coaches to support their players in RTS after ACLR [29]. These strategies were expressed by our athletes and also supported by previous literature. Other strategies that our athletes expressed that are, to our knowledge, not supported in the literature were that it may be helpful to discuss the training and match schedule with the athletes to identify appropriate time points for prevention training. Access to adequate rehabilitation facilities to perform alternative training (gym, cycling) alongside the pitch was important for our athletes.

The present study has both methodological strengths and limitations which needs to be considered when interpreting the results. Semi-structured interviews analyzed with inductive content analysis are suitable for describing informants experiences at a manifest level. Credibility was strengthened by the fact that the research group consisted of physiotherapists with clinical expertise of ACLR rehabilitation (AF, TG, SS), researchers within the field of ACL injuries (AF, SS) or qualitative research (AH, SS), and one medical student with own experience of ACLR and rehabilitation (NW). This provided an understanding of the patient group and the phenomena from various perspectives. There were no personal or professional relationships between the 2 interviewers and the athletes to ensure that the athletes could speak freely about their experiences and thoughts. The use of an interview guide and a thorough methodological path ensured that the findings represented the athletes’ perspectives of the phenomenon. The categorization process and the final categories were discussed among all authors to further increase credibility by investigator triangulation [23].

The 2 interviewers were a physiotherapist and a medical student (TG and NW), both with limited experience in qualitative research and interviewing. The use of an interview guide ensured that the same areas were asked for all athletes, thus increasing dependability [23]. Pilot interviews were conducted both to test the guide to increase dependability [23] and for the interviewers to gain experience in interviewing. Athletes were recruited up to 4 years after RTS which might affect trustworthiness because after such time the athletes might have difficulties to accurately describe their experiences of their finalphase rehabilitation and RTS process. The timeframe was chosen to ensure that the athletes have had time to go through rehabilitation and RTS after their ACLR, and that some time had passed without reinjury. Also in relation to the athletes´strategies to reduce reinjury risk, we strived to include athletes with varied time since RTS to get a wider perspective on athletes´ thoughts about reinjury. The age range was selected to reach the most common age group who injure their ACL, return to contact sports after ACLR and with a substantial risk for reinjury [1].

Findings from the present study may be transferable to other athletes with the same characteristics as the athletes in our study [23]. This study included a varied sample of 15 athletes with regards to age, sport, sport levels, sex, geographical locations, surgeons, self-reported knee function, emotions, confidence in performance, and risk appraisal in relation to RTS. Our sample had a larger proportion of women compared to the sex distribution of athletes undergoing ACLR in Sweden [33]. We aimed for recruiting 15–20 athletes based on a rather narrow aim, the specificity of the sample, and the method of analysis [34]. During the data collection process 15 athletes were deemed appropriate since the data collected was rich and the last interviews did not provide new knowledge related to the aim of the study. Choosing athletes with varied experiences also enriches the perspectives and deepens understanding of the phenomenon being researched, thus strengthening credibility of the findings [23].

## Conclusions

Athletes described strategies for a safe return to sport after ACL reconstruction, emphasizing continuous increased load, not forcing return to sport, injury prevention exercises, and seeking support from professionals and coaches. Despite loving their sport, the athletes had mixed feelings about undergoing additional rehabilitation if reinjured. The athletes recognized the high reinjury risk, attributing it to fate. These findings enhance understanding of athletes’ return to sport experiences after ACL reconstruction, their strategies to minimize reinjury risk, which might help optimizing care for this patient group.

### Electronic supplementary material

Below is the link to the electronic supplementary material.

Supplementary Material 1

Supplementary Material 2

## Data Availability

The datasets generated and/or analysed during the current study are not publicly available due to the decision and recommendations made by the Swedish Ethical Review Authority, but are available from the corresponding author on reasonable request.

## Abbreviations

ACL: Anterior cruciate ligament
ACL-RSI: ACL-Return to Sport after Injury scale
COREQ: Consolidated Criteria for Reporting Qualitative studies
IKDC-SKF: International Knee Documentation Committee Subjective Knee Evaluation Form
RTS: Return to sport
