# Supplementary material for: Athletes’ perspectives on return to sport after anterior cruciate ligament reconstruction and their strategies to reduce reinjury risk: a qualitative interview study
Source: BMC Sports Sci Med Rehabil. 2024 Jun 14;16:131. doi: 10.1186/s13102-024-00920-7 (PMC11177358; doi:10.1186/s13102-024-00920-7)
Supplement: Supplementary file 1 — Supplementary Material 1 [file 13102_2024_920_MOESM1_ESM.docx]

Athletes’ perspectives on return to sport after anterior cruciate ligament reconstruction and their strategies to reduce reinjury risk: a qualitative interview study

Supplementary material 1

**COREQ (COnsolidated criteria for REporting Qualitative research) 32 item Checklist**

Developed from:

*Tong A, Sainsbury P, Craig J. Consolidated criteria for reporting qualitative research (COREQ): a 32-item checklist for interviews and focus groups. Int J Qual Health Care. 2007 Dec;19(6):349-57.*

|  | **Item Number** | **Description** | **Reported on section or not applicable (N/A)** |
| --- | --- | --- | --- |
| ***Domain 1: Research team and reflexivity*** | | | |
| *Personal characteristics* | | | |
| Interviewer/  facilitator | 1 | Which author/s conducted the interview or focus group? | Data collection |
| Credentials | 2 | What were the researcher's credentials? *E.g. PhD, MD* | Data collection (TG an NW as interviewers)  Data analysis (AF,SS, AH) |
| Occupation | 3 | What was their occupation at the time of the study? | Data collection  Data analysis |
| Gender | 4 | Was the researcher male or female? | Data collection  Data analysis |
| Experience and training | 5 | What experience or training did the researcher have? | Information on pilot interviews in  Data collection  and information on the experience and training with regards to the researchers are provided in  Data analysis |
| *Relationships with participants* | | | |
| Relationship established | 6 | Was a relationship established prior to study commencement? | Data collection |
| Participant knowledge of the interviewer | 7 | What did the participants know about the researcher? *E.g. Personal goals, reasons for doing the research* | Data collection |
| Interviewer characteristics | 8 | What characteristics were reported about the interviewer/facilitator? *E.g. Bias, assumptions, reasons and interests in the research topic* | Data collection |
| ***Domain 2: Study design*** | | | |
| *Theoretical framework* | | | |
| Methodological orientation and theory | 9 | What methodological orientation was stated to underpin the study? *E.g. grounded theory, discourse analysis, ethnography, phenomenology, content analysis* | Study design  Data analysis  Discussion |
| *Participant selection* | | | |
| Sampling | 10 | How were participants selected? *E.g. purposive, convenience, consecutive, snowball* | Participants and setting |
| Method of approach | 11 | How were participants approached? *E.g. face-to-face, telephone, mail, email* | Participants and setting |
| Sample size | 12 | How many participants were in the study? | Results |
| Non-participation | 13 | How many people refused to participate or dropped out? What were the reasons for this? | Data analysis |
| *Setting* |  |  |  |
| Setting of data collection | 14 | Where was the data collected? *E.g. home, clinic, workplace* | Participants and setting |
| Presence of nonparticipants | 15 | Was anyone else present besides the participants and researchers? | Data collection |
| Description of sample | 16 | What are the important characteristics of the sample? *E.g. demographic data, date* | Participants and setting  Results  Discussion |
| *Data collection* | | | |
| Interview guide | 17 | Were questions, prompts, guides provided by the authors? Was it pilot tested? | Data collection |
| Repeat interviews | 18 | Were repeat interviews carried out? If yes, how many? | n/a |
| Audio/visual recording | 19 | Did the research use audio or visual recording to collect the data? | Data collection |
| Field notes | 20 | Were field notes made during and/or after the interview or focus group? | Data collection |
| Duration | 21 | What was the duration of the interviews or focus group? | Data collection |
| Data saturation | 22 | Was data saturation discussed? | Data collection |
| Transcripts returned | 23 | Were transcripts returned to participants for comment and/or correction? | No, n/a |
| **Domain 3: analysis and findings** | | | |
| *Data analysis* | | | |
| Number of data coders | 24 | How many data coders coded the data? | Data analysis for authors responsibilities in the Data analysis |
| Description of the coding tree | 25 | Did authors provide a description of the coding tree? | Data analysis |
| Derivation of themes | 26 | Were themes identified in advance or derived from the data? | Coding and categorization process  Data analysis |
| Software | 27 | What software, if applicable, was used to manage the data? | Data analysis |
| Participant checking | 28 | Did participants provide feedback on the findings? | No, n/a |
| *Reporting* | | | |
| Quotations presented | 29 | Were participant quotations presented to illustrate the themes / findings? Was each quotation identified? *E.g. Participant number* | Quotations provided throughout the Results. Informant numbers included |
| Data and findings consistent | 30 | Was there consistency between the data presented and the findings? | Consistency of findings throughout the analysis process: Coding tree Data analysis  Presentation of main categories and sub-categories in Table 3  Results  Findings presented in the text of the Results |
| Clarity of major themes |  | Were major themes clearly presented in the findings? | Main categories are described in the Results |
| Clarity of minor themes | 32 | Is there a description of diverse cases or discussion of minor themes? | Sub-categories are described in the Results |
