# Supplementary material for: Athletes’ perspectives on return to sport after anterior cruciate ligament reconstruction and their strategies to reduce reinjury risk: a qualitative interview study
Source: BMC Sports Sci Med Rehabil. 2024 Jun 14;16:131. doi: 10.1186/s13102-024-00920-7 (PMC11177358; doi:10.1186/s13102-024-00920-7)
Supplement: Supplementary file 2 — Supplementary Material 2 [file 13102_2024_920_MOESM2_ESM.docx]

Athletes’ perspectives on return to sport after anterior cruciate ligament reconstruction and their strategies to reduce reinjury risk: a qualitative interview study

Supplementary material 2

# Interview guide

## Return to sports

Tell me (in broad terms) how your rehabilitation after the ACL reconstruction was

- How much contact did you have with physiotherapist?
- Where did you do your rehabilitation?
- Was your physiotherapist a ”sports physiotherapist”?
- When did you start/end your rehabilitation?
- When did you start attending your team's practices?
- Can you tell me what the arrangement was like regarding the rehabilitation phase when you started to join your team?
- Can you tell me what the rehabilitation was like from participating in training to participating in a match?

Can you tell me about your experiences of the rehabilitation in the return to sport phase?

How did you feel about your return to training?

- Can you tell me more about your thoughts AND feelings about return to training.

Can you describe your experiences of returning to competition/matches?

## Thoughts about reinjury

Have you thought about the risk of a reinjury to one of your knees?

- If yes, how did the thought of a reinjury affect your decision/feelings/behaviour about returning to sport?

## How to prevent reinjury?

If you think about the different actors involved in the prevention of reinjury, i.e. the athlete, the orthopaedic surgeon, the physiotherapist and the coach; what can these actors do to prevent reinjuries?

Follow-up questions (if not stated in the answer):

- What do you think about the possibility of reducing the risk of reinjuries
- Do you do anything to prevent reinjury?

According to your experiences, how do the efforts of the various actors fulfil the expectations you have of preventive work?

- Describe expectations and in what way the actors' efforts corresponded/did not correspond to your expectations.

If you had unlimited resources to deploy interventions/supports (in addition to what it looks like today) to prevent reinjuries, what interventions would you prefer?

**Other comments**

Are there additional aspects of what we discussed that you think we should highlight? Would you like to add anything you think is important?

Thank you for your participation.

**Follow-up questions:**

- What do you mean by….?
- Can you elaborate…?
- Can you give examples of...?
- Have I understood correctly...?
